# Epidemiology of human Monkey-pox cases in Rivers State, Nigeria January 2017-June 2022

**DOI:** 10.3389/fpubh.2022.1039604

**Published:** 2023-01-04

**Authors:** Hastings Chinedu Onu, Owhonda Golden, I. Aaron Wali, B. Elizabeth Adedire, Muhammed Balogun, Eze Chidinma, S. Adebowale Ayo, Okolocha Emmanuel

**Affiliations:** ^1^Nigeria Field Epidemiology and Laboratory Training Program, Abuja, Nigeria; ^2^Department of Public Health and Disease Control, Rivers State Ministry of Health, Port Harcourt, Rivers State, Nigeria; ^3^Department of Community Medicine, Rivers State University, Port Harcourt, Rivers State, Nigeria; ^4^African Field Epidemiology Network, Abuja, Nigeria; ^5^School of Public Health, University of Port Harcourt, Port Harcourt, Rivers State, Nigeria; ^6^Department of Epidemiology and Medical Biostatistics, University of Ibadan, Ibadan, Oyo State, Nigeria; ^7^Department of Veterinary Public Health and Preventive Medicine, Ahmadu Bello University, Zaria, Kaduna, Nigeria

**Keywords:** monkey-pox, epidemiology, Rivers State, Nigeria, trend, distribution, surveillance

## Abstract

The resurgence in monkey pox disease has posed a global health threat. Nigeria recorded increased number of reported monkey pox cases in 2017, with cases occurring in subsequent years. Notably, cases of monkey pox had been reported in western countries from an epidemiologically linked traveler with a travel history to Nigeria. The highest burden of cases of monkey pox is recorded in River state, Nigeria. Consequently, there is the need to examine the epidemiology of monkey pox according to time, place, person and geography in Rivers state. A retrospective analysis of monkey pox cases was conducted based on the data obtained from the Rivers State Disease Surveillance and Notification unit from January 2017-June 2022. A total of 112 suspected cases were reported during this period of which 49 (44%) were confirmed by laboratory diagnosis. Obio-Akpor (29) and Port Harcourt (9) Local Government Areas which make up the urban centers of the state recorded most cases of monkey pox. More males (36) were affected than females (10), and the age group mostly affected was from 20 to 29 years, however increased cases of monkey pox was found in the months of September to November in most of the years reviewed. Monkey pox is prevalent in Rivers state, although the pattern follows a declining trend. Therefore, the state government should put appropriate mechanisms in place to eradicate the disease.

## Introduction

Monkey-pox is a viral zoonotic disease characterized by fever and malaise accompanied by progressive appearance of vesiculopustular skin lesions (1). Although, it is quite similar to presentation; they are epidemiologically distinct disease (2). The symptoms can last from two to 4 weeks (1, 2). However, severe cases often occur in children and the immune-compromised individuals. Monkey pox virus is mostly transmitted to people from wild animals - such as rodents, and other primates such as monkeys; Human-to-human transmission occurs, through contact with lesions on the skin or on internal mucosal surfaces of an infected person, such as mouth or throat, respiratory droplets, body fluids, and also from fomites like contaminated materials, beddings, and other objects (3). However, cases of sexual transmission of the disease have been documented (4).

Since 1 January 2022 to 22 June 2022, there has been 3,413 laboratory confirmed cases of monkey-pox with one death reported from 50 countries/territories in five WHO Regions (5, 6). The majority of these confirmed cases (86%) were reported from the European Region, while regions in America (11%), Eastern Mediterranean (< 1%), Western Pacific (< 1%), and Africa (2%) have reported cases of monkey pox (7). Nigeria recorded its first case of monkey pox in 1971 and subsequently recorded a 3^rd^ case in 1978. There was no report of monkey pox cases for about 40 years, until 2017 when 197 cases were recorded in 23 out of the 36 states in the country (8–10). Thus, monkey pox is classified as one of the epidemic-prone diseases that require immediate reporting under the Integrated Disease Surveillance and Response system (IDSR).

Rivers State has recorded five cases of monkey pox in 2022 and which 49 (44%) were confirmed by laboratory diagnosis. Rivers State has the highest number of monkey-pox from September 2017 to June 2022 in Nigeria (11), The identify, isolate, inform framework is adopted by the ministry of health in reducing the risk of transmission in health care settings and has been applied broadly, Once identified, clinicians need to ensure the patient with the suspected infection of interest is appropriately isolated. The Rivers State ministry of Health operates the operation emergency system which optimizes the collaboration of the different pillars, the Risk communication, the laboratory, also the surveillance and point of entry pillars which are all activated following outbreaks.

This study was carried out to determine the epidemiology of human monkey pox in Rivers State from 2017 to 2022.

## Method

### Study settings

Rivers State is in tropical rainforest with mangrove swamps and rivers. Rainfall is generally seasonal and occurs between the months of March through November and peaks in July. Due to its geographical location, Rivers State hosts a vast array of wildlife and plants species (12). The State has vast reserves of crude oil and natural gas, thus, careers for the urban dwellers are predominantly oil exploration, engineering, and fish farming. Transportation systems are *via* land, seaport, and an international airport. Therefore, the state has an exposure to international travelers. Being one of the urbanized cities in Nigeria with enormous trading activities, the state attracts migrants from other states in Nigeria and suburb neighboring countries. Rivers State has 23 Local Governments Areas with a projected population of about 8,657,375 as at mid-year 2022 (7).

### Study design

Retrospective secondary data analysis of the human monkey pox surveillance data collected in Rivers State from January 2017 to June 2022.

### Study population

The data analyzed was obtained through a population-based surveillance system implemented in all the LGAs of Rivers State.

### Surveillance system in Nigeria

Monkey pox data is generated through the integrated disease surveillance response system in Nigeria. Monkey pox surveillance in Rivers State is through the IDSR platform, a national disease reporting platform, covering priority diseases from all health facilities across the nation. The monkey pox IDSR data was obtained from the Surveillance Unit of Rivers State Ministry of Health. The information flows from the health facilities, through the Ward Focal persons to the Local Government Areas (LGAs) Disease Surveillance and Notification officers (DSNOs), to the States DSNOs. The IDSR collects information on monkey pox cases and deaths, facility location, and laboratory outcomes.

### Monkey pox case definitions

A suspected case was defined as any person presenting with a history of sudden onset of fever, followed by a pustular rash occurring mostly on the face, palms and soles of feet.

A probable case was defined as any suspected case with epidemiological linkage with a confirmed case in which laboratory testing could not be carried out.

A confirmed case was defined as any suspected case with laboratory confirmation (positive IGM antibody or virus isolation). Positive PCR alone is suggestive of a confirmed case of independent of IGM results (13).

Data were analyzed using descriptive statistics and findings were presented in charts and tables. The trend in monkey pox cases was assessed using linear regression model, *y*_*i*_ = *a*+*bt*_*i*_+ε_*i*_; where a and b are constant parameters of the regression model. The dependent variable, y_i_ is the number of monkey pox cases, t_i_ is the time which denotes the independent variable. The error term is ε_*i*_.

### Ethical considerations

A protocol for this study was developed and submitted to the Ethics Committee of the Rivers State Ministry of Health and the permission to conduct this study was granted subsequently. The investigators had no contact with the cases as secondary data was used,no personal information was presented and all information in the data were annonymised.

## Results

The number of suspected Monkey-pox cases was 112 out of which 46 (46.7%) cases were confirmed by RT-PCR in the laboratory. The mean age of confirmed cases was 33 years (range 2–49) and 72.7% were males.The overall Case Fatality Rate (CFR) was 1.8% for the period under review. Forty one (38.0%) of the monkey pox cases were in the age group 20–39 years which constituted the highest proportion, while the least, 9 (8.3%) were among the age group 10–19 years.

**Figure 1:**
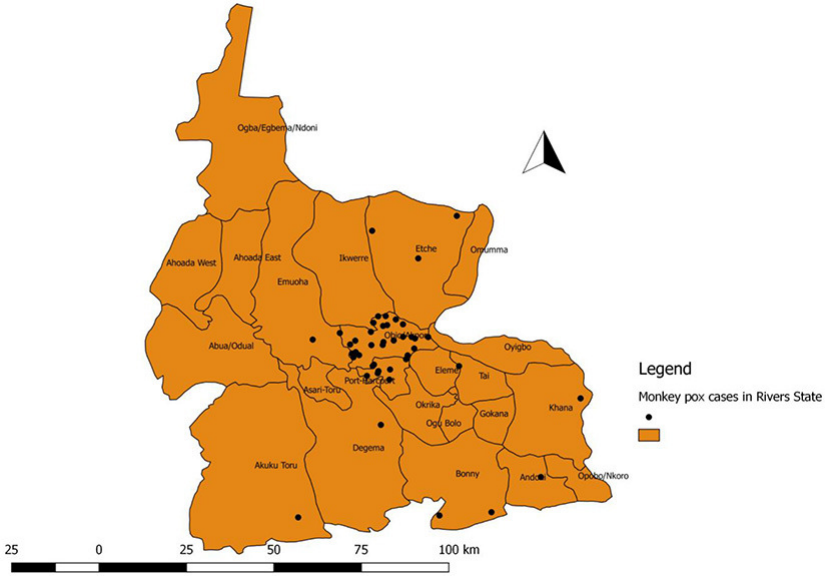
Distribution of confirmed (positive) cases of Monkey pox in the Local Government Areas of Rivers State, Nigeria, January 2017-June 2022.

**Figure 2:**
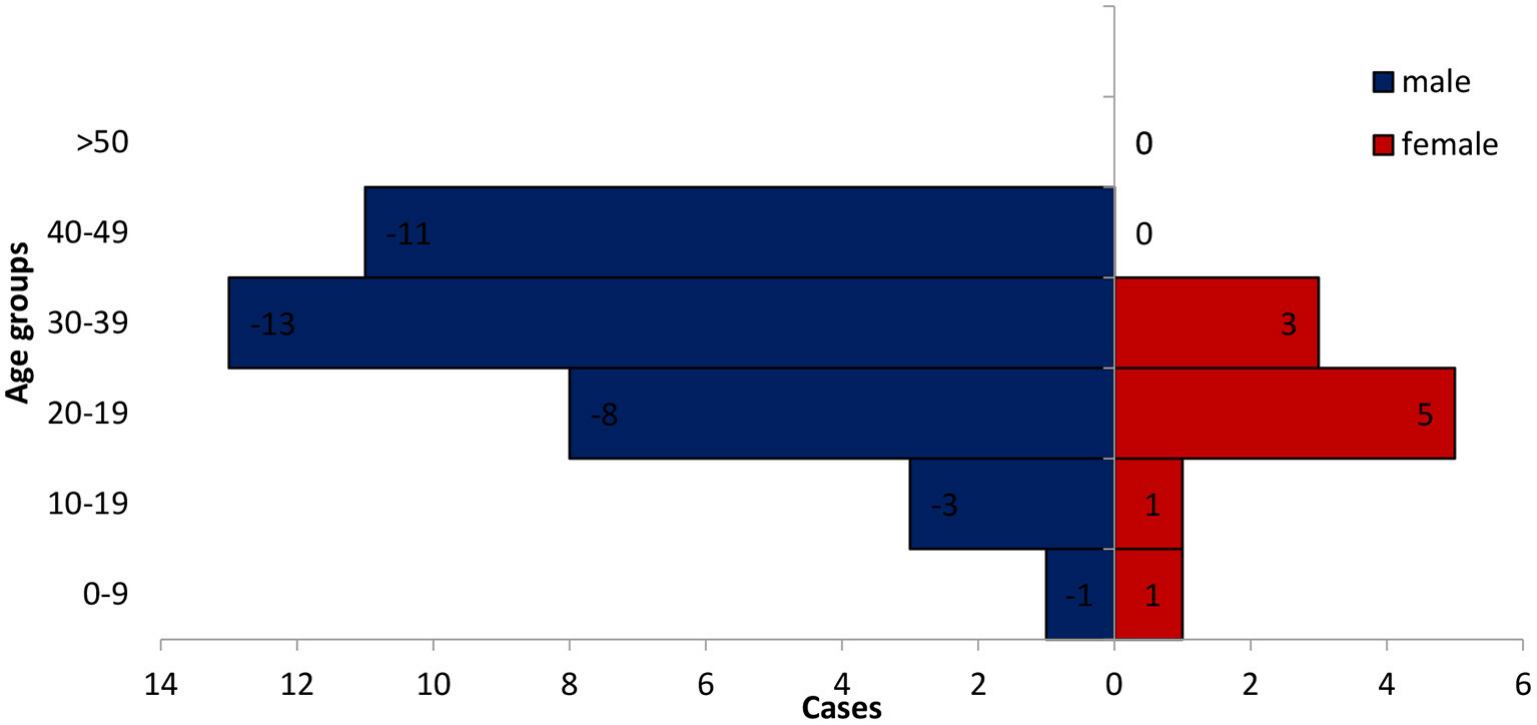
Age-sex distribution of confirmed cases of Monkey-pox in Rivers State, Nigeria January 2017-June 2022.

**Figure 3:**
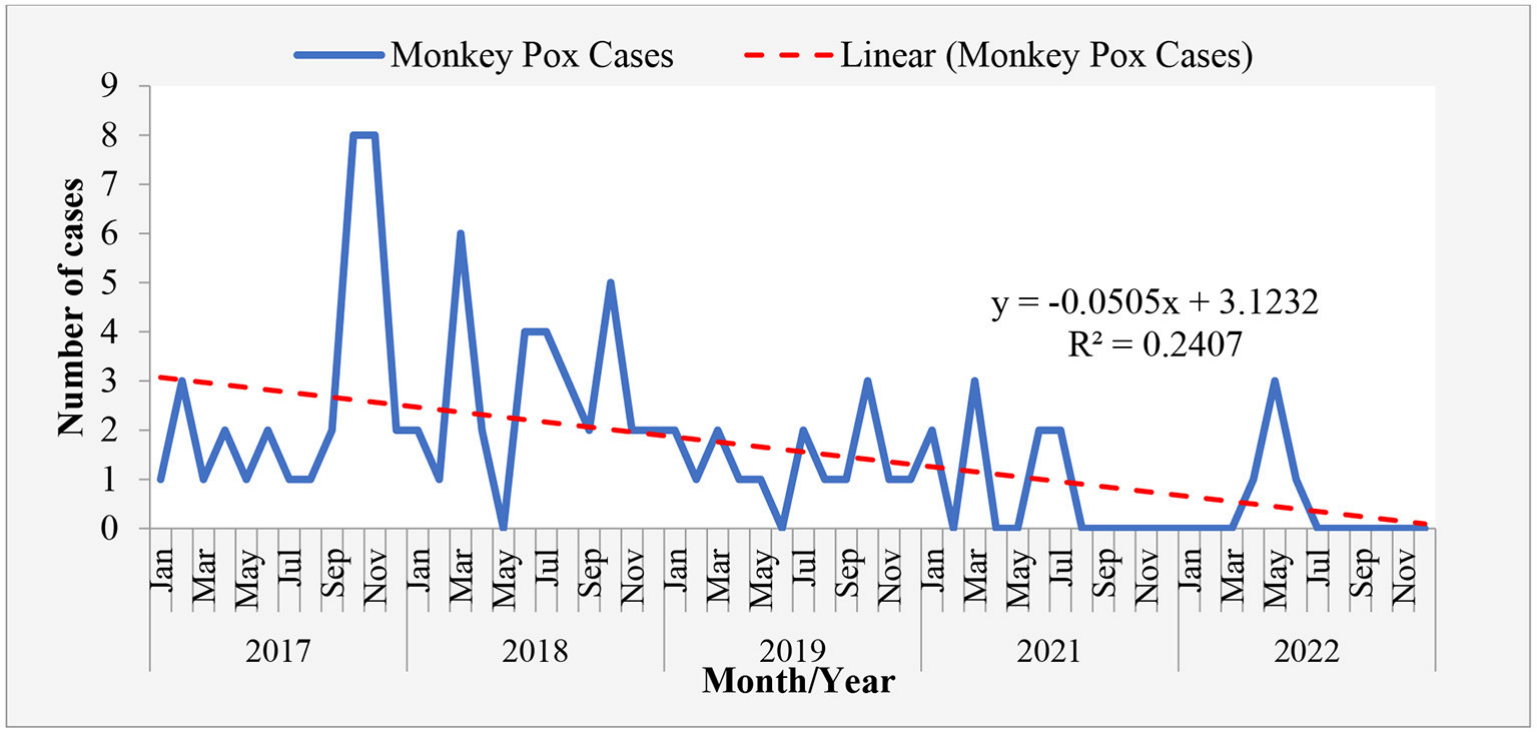
Trend in Monkey-pox cases in Rivers State January 2017-June 2022.

The monthly reported cases Revealed that the cases of monkey pox in Rivers State follows a downward trend with reduction rate being −0.0505. It shows reduction in monkey pox cases as the data progress in month (Table 1, Figures 1–3).

**Table 1:** Sociodemographic characteristics of suspected Monkey-pox cases in Rivers State january 2017-june 2022.

**Age**	**(n)**
>10	16
10–19	9
20–29	41
30–39	28
40–49	15
50 <	3
**Sex**	
Male	80
Female	30
Unknown	2
**Year**	
2017	33
2018	36
2019	18
2020	0
2021	20
2022	5
**Outcome**	
Alive	109
Dead	2
Unknown	1

## Discussion

Monkey pox is still a problem of an important concern in Nigeria as more cases are being reported on daily basis and Rivers state is an epicenter for the disease. In Rivers State, more cases of Monkey-pox had been cumulatively reported from January 2017 to June 2022 than any other states in Nigeria (14). The mangrove swamps, rainforest vegetation, and proximity of the state to both local and international travelers can explain the upsurge in monkey pox in Rivers state (8, 14, 15). Other states in the south south region in Nigeria which have similar vegetation to that of Rivers state also experienced high number of monkey pox cases (16). The finding corroborates what has been established in the literature where higher number of Monkey-pox cases were reported in the southern than the northern regions in Nigeria (17, 18).

The study showed that less than half of the suspected cases (by case definition) were confirmed by RT-PCR. There was a higher incidence of Monkey-pox in Obio-Akpor and Port-Harcourt City Local Government Areas in Rivers state. These two LGAs are predominately urban areas but rural dwellers are also visible at these LGAs on daily basis for trading and business activities. Although, Monkey-pox is known as a disease of rural rainforests where there are more contacts with bush meat (19). However, the rural dwellers interaction with the urban residence is in large numbers and the rural-urban migration is prevalent across the state These urban centers are more populated (make up to about 30% of the total population of the State) and may be suggestive of endemicity and changes in epidemiology of Monkey-pox (19–21).

That the study further revealed that males were more infected with monkey pox in Rivers state than the females. This finding corroborates the outcome of recent studies that shows that more monkey pox infections occur in males than females (16, 22). Higher reported cases of monkey pox among males than females can be explained by their proximity to job activities outside their homes. For instance, the males are more involved in the gas and oil exploration activities in which their careers predispose them to locations in the rainforest vegetation, where exposures to are more likely (23), nevertheless this does not rule out human to human transmission of the diseases although there is no evidence that human to human transmission can sustain an outbreak (9). This contradicts other studies which showed that Monkey-pox occurs equally in male as in females (15).

The finding that more cases of Monkey-pox among persons aged 20–40 years which are active youths corroborates other studies that found persons within the same age-range reporting more cases of Monkey-pox (17, 22). Early studies found that more Monkey-pox cases were reported more in children between the ages of 3–4 years and 5–6 years this differs from our study (24, 25). The termination of routine smallpox vaccination globally about 40 years ago after the eradication of smallpox may have been a contributory factor to incidence of monkey-pox among persons below 40 years of age (21).

The months of September and November, in which we have noticed an increase in the number of reported cases fall in these season when flooding occurs and brings human and Monkey-pox infected animals together (18). The trend line, however, shows a decline in Monkey-pox cases from 2017 to 2022, notwithstanding there was no recorded case in 2020. This could be linked to the overwhelming effect of the COVID-19 pandemic on the routine surveillance system (26, 27). Being a disease of contact transmission, the social distancing and lock downs would have helped in reducing transmission of Monkey-pox during the COVID-19 pandemic. The case fatality rate of monkey pox in the reviewed years is 4%.

## Limitations

The missing data from review data of Monkey-pox did not allow an elaborate analysis of indicators of interest like travel history and symptoms of Sexual transmission of monkey-pox disease which are reported from other western countries.

## Conclusion and recommendation

This study shows that between 2017 and 2022 more cases of Monkey-pox disease were identified in Port Harcourt and Obio-Akpor LGA and more commonly occurred in September and November affecting mostly 20–40 years old, the missing data didn't provide adequate knowledge on the sexual transmission infections which has been documented in western countries therefore health workers and surveillance officers need to conduct proper outbreak investigation, and surveillance so as to enable more knowledge on the secondary attack rate for Monkey-pox, consequently provide more information on the risk factors for monkey pox.

## Data availability statement

The data analyzed in this study is subject to the following licenses/restrictions: Data can only be assessed on application to the Rivers State Ministry of Health. Requests to access these datasets should be directed to https://rsmoh.riversstate.gov.ng/.

## Ethics statement

Written informed consent was obtained from the individual(s), and minor(s)' legal guardian/next of kin, for the publication of any potentially identifiable images or data included in this article.

## Author contributions

HO contributed to the conception, analysis and design of the works, and final corrections of the works prior to submission. IW and OG supervised the conception of the work. BA, MB, and OE were reviewers of the all the works. EC and SA supervised the data analysis. All authors contributed to the works and approved the submitted version.
